# Supplementary material for: Joint cell segmentation and cell type annotation for spatial transcriptomics
Source: Mol Syst Biol. 2021 May 31;17(6):e10108. doi: 10.15252/msb.202010108 (PMC8166214; doi:10.15252/msb.202010108)
Supplement: Supplementary file 1 — Expanded View Figures PDF [file MSB-17-e10108-s006.pdf]

Expanded View Figures

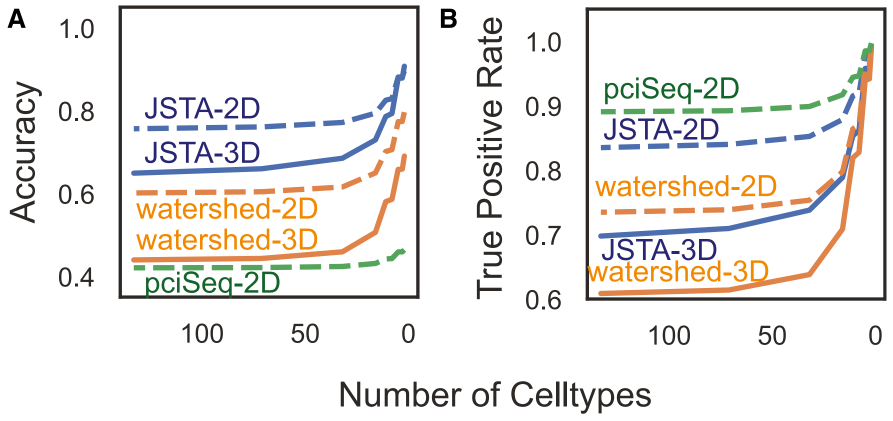

Figure EV1. Performance evaluation of JSTA, pciSeq, and watershed.

A, B pciSeq is unable to run on 3D data (solid line), so we simulated additional 2D data (dotted line). We evaluated these methods on the performance of accuracy of assigning mRNA to the correct cell (A). JSTA is more accurate than pciSeq on the accuracy metric. pciSeq is not very accurate here, because many mRNA are incorrectly assigned to background. We additionally tested these methods on their performance of assigning mRNA to the correct cell while ignoring mRNA assigned to background (B). pciSeq is highlighted here, because it mainly assigns spots close to the nucleus; JSTA is comparable.

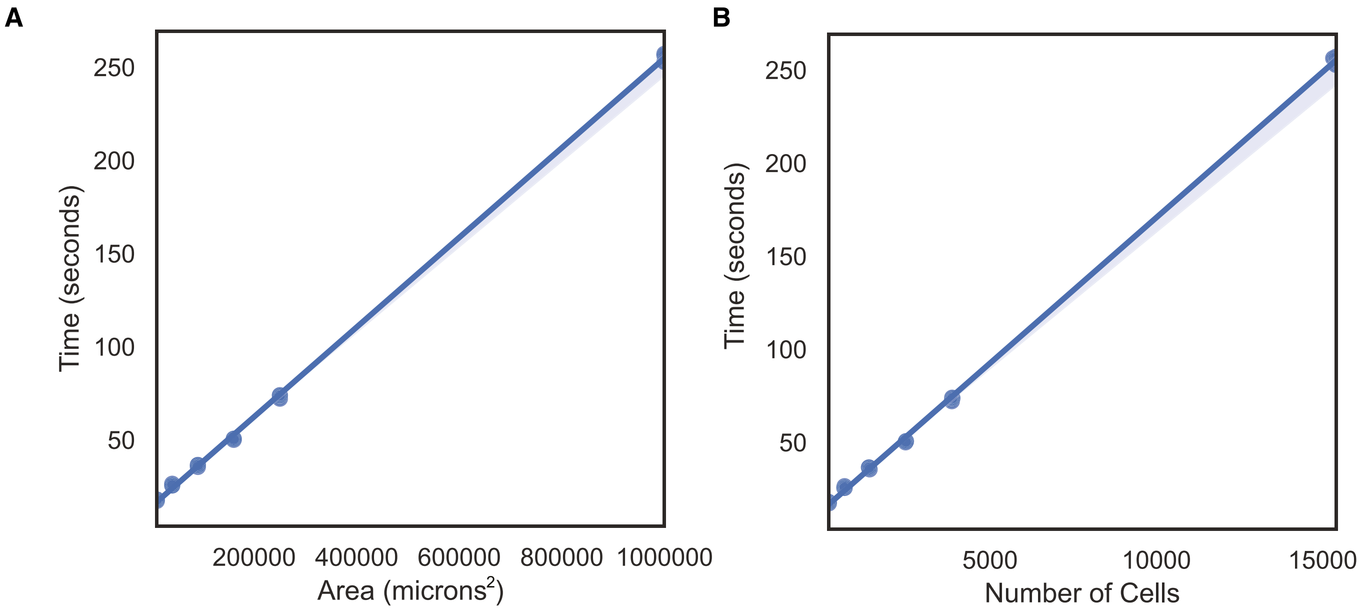

Figure EV2. Application of JSTA to osmFISH data from the mouse somatosensory cortex.

A Glutamatergic neurons are consistent with previously identified spatial patterns of the somatosensory cortex.

B JSTA-mapped high-resolution (sub)types are correlated with their NCTT counterparts in terms of gene expression patterns (Table EV4). Cell types with at least five cells were kept.

Figure EV3. Run time evaluation of JSTA on simulated data.

A, B We ran JSTA on data simulated with a width and height of 100, 200, 300, 400, 500, and 1,000 μm, with three replicates each. We evaluated the time taken to run JSTA by the area of the section (A), and the number of cells in each section (B).

▶

 © 2021 The Authors

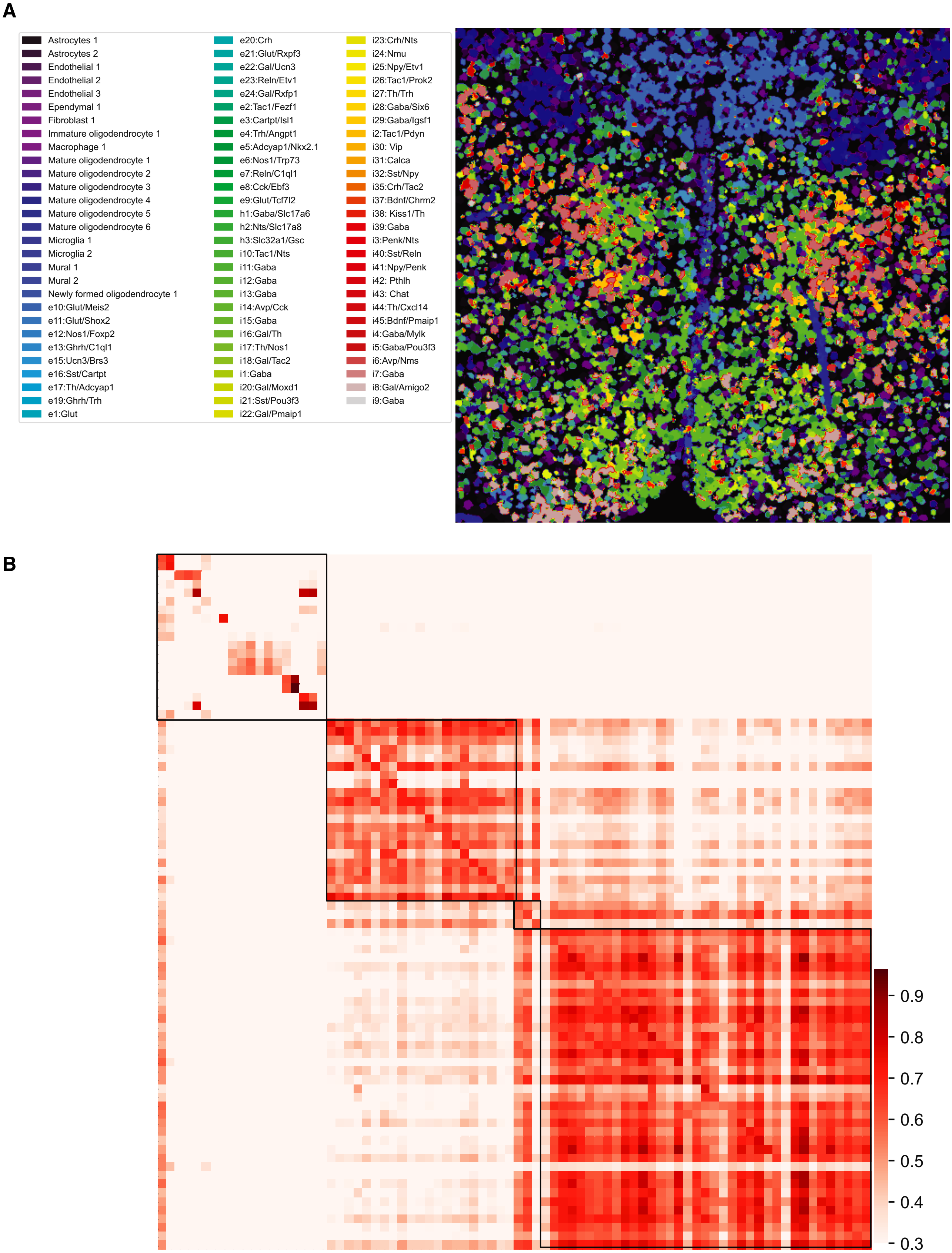
A
Astrocytes 1
Astrocytes 2
Endothelial 1
Endothelial 2
Endothelial 3
Ependymal 1
Fibroblast 1
Immature oligodendrocyte 1
Macrophage 1
Mature oligodendrocyte 1
Mature oligodendrocyte 2
Mature oligodendrocyte 3
Mature oligodendrocyte 4
Mature oligodendrocyte 5
Mature oligodendrocyte 6
Microglia 1
Microglia 2
Mural 1
Mural 2
Newly formed oligodendrocyte 1
e10:Glut/Meis2
e11:Glut/Shox2
e12:Nos1/Foxp2
e13:Ghrh/C1ql1
e15:Ucn3/Brs3
e16:Sst/Cartpt
e17:Th/Adcyap1
e19:Ghrh/Trh
e1:Glut
e20:Crh
e21:Glut/Rxpf3
e22:Gal/Ucn3
e23:Reln/Etv1
e24:Gal/Rxfp1
e2:Tac1/Fezf1
e3:Cartpt/Isl1
e4:Trh/Angpt1
e5:Adcyap1/Nkx2.1
e6:Nos1/Trp73
e7:Reln/C1ql1
e8:Cck/Ebf3
e9:Glut/Tcf7l2
h1:Gaba/Slc17a6
h2:Nts/Slc17a8
h3:Slc32a1/Gsc
i10:Tac1/Nts
i11:Gaba
i12:Gaba
i13:Gaba
i14:Avp/Cck
i15:Gaba
i16:Gal/Th
i17:Th/Nos1
i18:Gal/Tac2
i1:Gaba
i20:Gal/Moxd1
i21:Sst/Pou3f3
i22:Gal/Pmaip1
i23:Crh/Nts
i24:Nmu
i25:Npy/Etv1
i26:Tac1/Prok2
i27:Th/Trh
i28:Gaba/Six6
i29:Gaba/Igsf1
i2:Tac1/Pdyn
i30: Vip
i31:Calca
i32:Sst/Npy
i35:Crh/Tac2
i37:Bdnf/Chrm2
i38: Kiss1/Th
i39:Gaba
i3:Penk/Nts
i40:Sst/Reln
i41:Npy/Penk
i42: Pthlh
i43: Chat
i44:Th/Cxcl14
i45:Bdnf/Pmaip1
i4:Gaba/Mylk
i5:Gaba/Pou3f3
i6:Avp/Nms
i7:Gaba
i8:Gal/Amigo2
i9:Gaba
B
0.9
0.8
0.7
0.6
0.5
0.4
0.3

Figure EV3.

Figure EV4. Application of JSTA to MERFISH data from the mouse hypothalamic preoptic region.

A High-resolution cell types identified by JSTA. The spatial mappings of these high-resolution cell types are consistent with the manually annotated data from Moffit *et al* (2018).

B JSTA-mapped high-resolution (sub)types are highly correlated with their scRNAseq reference counterparts in terms of gene expression patterns (Table EV3). Cell types with at least five cells were kept.

© 2021 The Authors

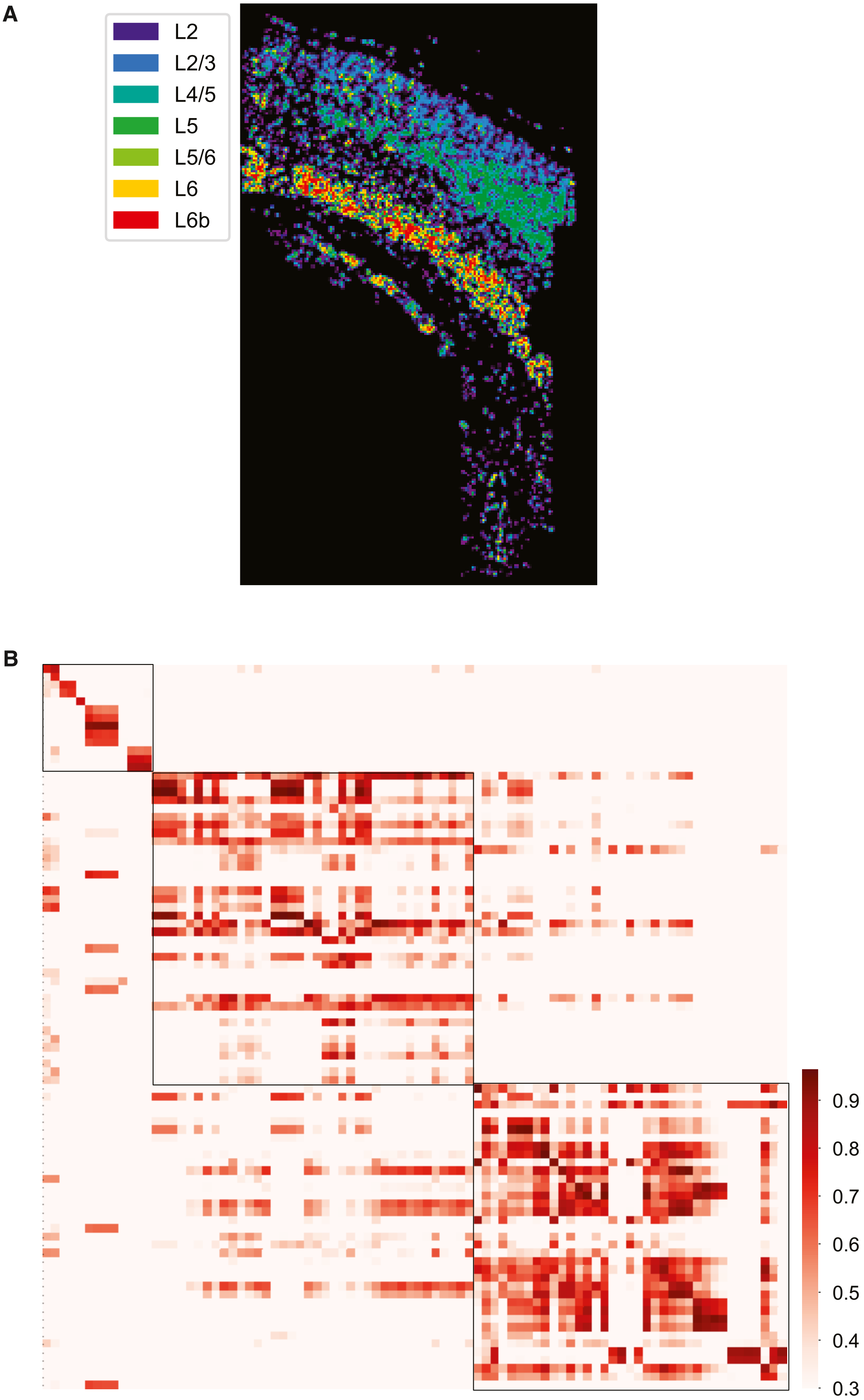

Figure EV4.

© 2021 The Authors

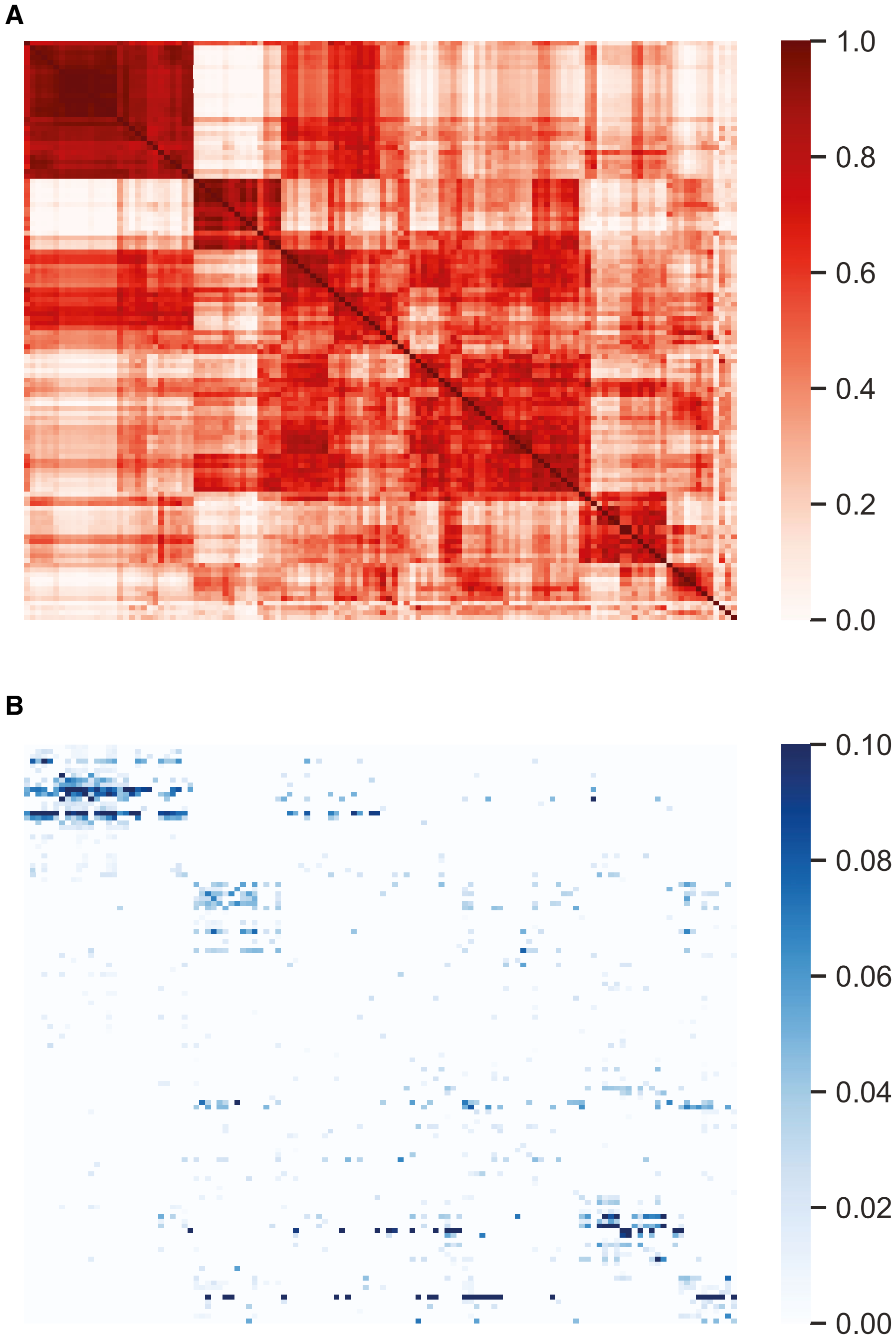

Figure EV5. Correlation structure of cell types compared with their colocalization.

Neuronal subtypes that are highly colocalized are often correlated in their gene expression. Cell types with more than 10 cells were included.

A Pearson correlation of 122 (sub)types across 83 selected genes.

B Frequency of neighbors between each of 122 (sub)types. Only significant (FDR < 0.05) colocalizations are shown. Labels and values are detailed in Tables EV5 and EV6.

Figure EV6. Identification of spatial differentially expressed genes (spDEGs).

spDEGs were computed by comparing the true variance in gene expression between cell subtype neighborhoods to that of randomly permuted cell (sub)type neighborhoods.

A 63 genes across 61 cell types show significant spDEGs. Heatmap values correspond to $-\log_2$(*P*-value).

B Number of spDEGs in each of the 61 cell types.

C Number of cell types with each of the 63 spDEGs.

 © 2021 The Authors

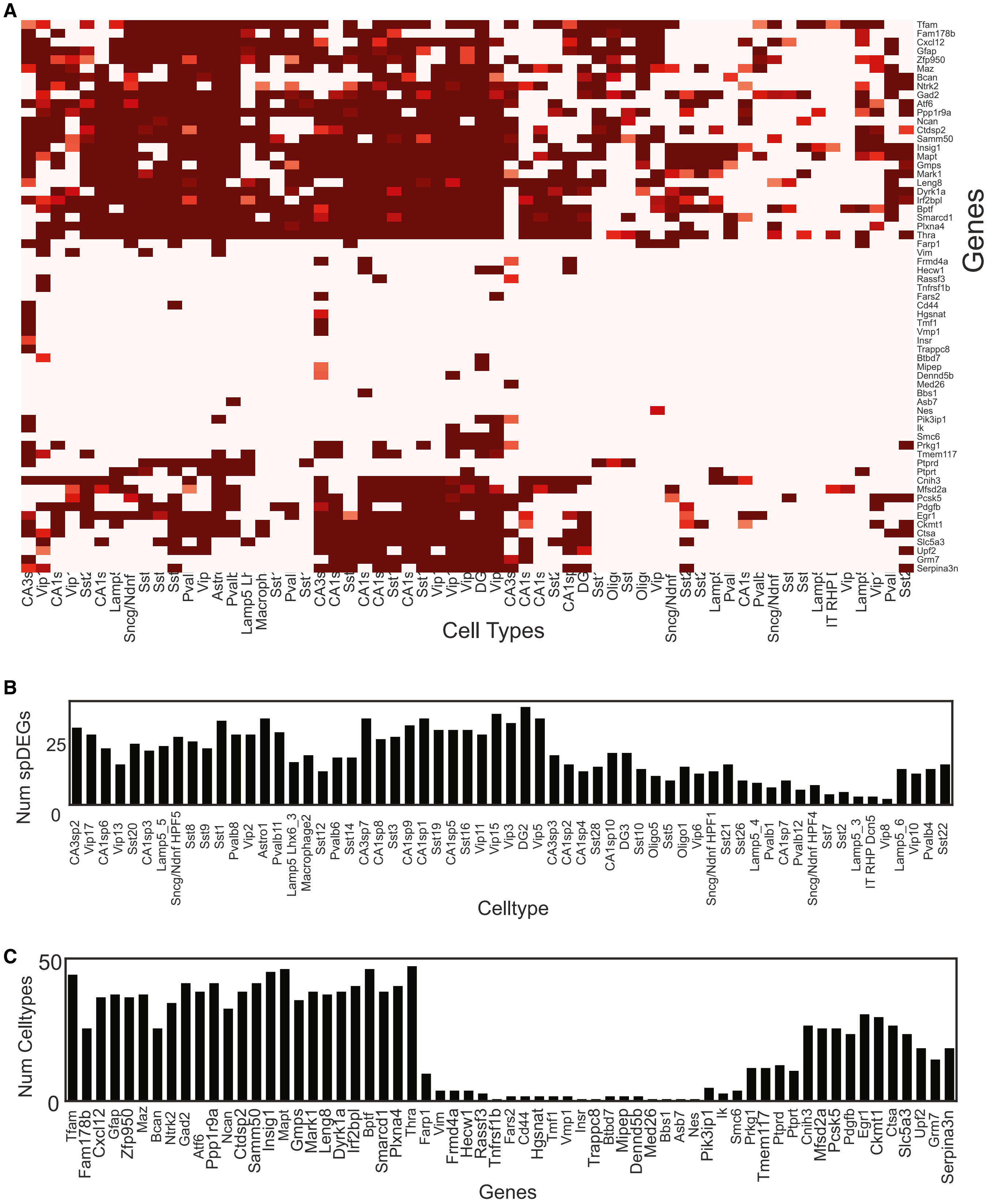
A
Genes
Cell Types
B
Num spDEGs
Celltype
C
Num Celltypes
Genes

Figure EV6.

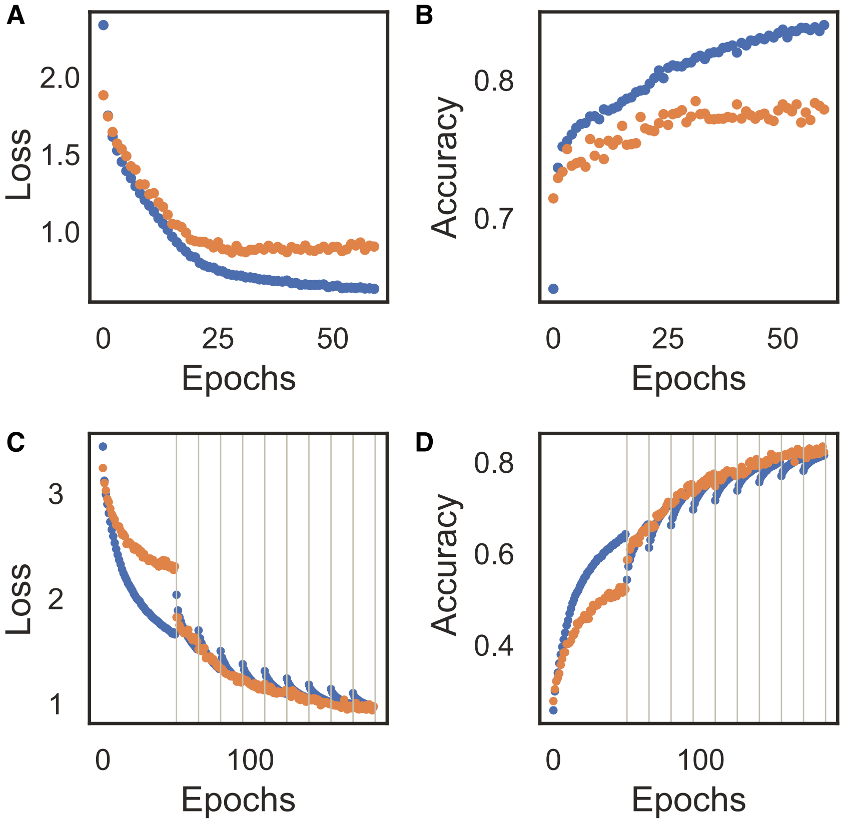

Figure EV7. Cross-entropy loss and accuracy of cell type (A, B) and pixel (C, D) classifier during training for the train (blue) and validation (orange) datasets.

A, B Cross-entropy (A) loss and accuracy (B) during training cell type classifier. The cell type classifier overfits the training data and is mitigated by stopping training after 40 epochs.

C, D Cross-entropy loss (C) and accuracy (D) during training of the pixel classifier. Black lines indicate new training iteration after pixel reassignment.

 © 2021 The Authors
