# Supplementary material for: Joint cell segmentation and cell type annotation for spatial transcriptomics
Source: Mol Syst Biol. 2021 May 31;17(6):e10108. doi: 10.15252/msb.202010108 (PMC8166214; doi:10.15252/msb.202010108)
Supplement: Supplementary file 2 — Table EV1 [file MSB-17-e10108-s007.docx]

**Table EV1**

| Layer Type | Num Nodes | Activation | Regularization |
| --- | --- | --- | --- |
| Input | 83 | - | - |
| Dense | 249 | tanh | L1 (5e^-3^) |
| Batch Normalization | - | - | - |
| Dense | 249 | tanh | L1 (5e^-3^) |
| Batch Normalization | - | - | - |
| Output | 133 | softmax | L1 (5e^-3^) |

Table EV1. Cell type classifier architecture. The network was initialized with Xavier initialization. Learning rates of 5e^-3^ and 5e^-4^ for 20 epochs each. A batch size of 64 was used. The Adam optimizer was used to update parameters. Cross entropy loss was used.
