# Supplementary material for: Joint cell segmentation and cell type annotation for spatial transcriptomics
Source: Mol Syst Biol. 2021 May 31;17(6):e10108. doi: 10.15252/msb.202010108 (PMC8166214; doi:10.15252/msb.202010108)
Supplement: Supplementary file 3 — Table EV2 [file MSB-17-e10108-s001.docx]

**Table EV2**

| Layer Type | Num Nodes | Activation | Regularization |
| --- | --- | --- | --- |
| Input | 83 | - | - |
| Dense | 166 | tanh | L1 (1e^-3^) |
| Batch Normalization | - | - | - |
| Dense | 166 | tanh | L1 (1e^-3^) |
| Batch Normalization | - | - | - |
| Dense | 332 | tanh | L1 (1e^-3^) |
| Batch Normalization | - | - | - |
| Output | 133 | softmax | L1 (1e^-3^) |

Table EV2. Pixel classifier architecture. The network was initialized with Xavier initialization. Initially the model was trained for 25 epochs with 1e^-4^ and 1e^-3^ learning rate. Subsequent updates were done on 15 epochs with a learning rate of 1e^-4^. We used the Adam optimizer to update parameters. Cross entropy loss was used.
